# Smartphone Applications in Dentistry: A Scoping Review

**DOI:** 10.3390/dj11100243

**Published:** 2023-10-20

**Authors:** Maurizio Pascadopoli, Paolo Zampetti, Maria Gloria Nardi, Matteo Pellegrini, Andrea Scribante

**Affiliations:** 1 Unit of Orthodontics and Pediatric Dentistry, Section of Dentistry, Department of Clinical, Surgical, Diagnostic and Pediatric Sciences, University of Pavia, 27100 Pavia, Italy; maurizio.pascadopoli01@universitadipavia.it (M.P.); paolo.zampetti@unipv.it (P.Z.); andrea.scribante@unipv.it (A.S.); 2 Maxillofacial Surgery and Dental Unit, Fondazione IRCCS Cà Granda Ospedale Maggiore Policlinico, 20122 Milan, Italy; 3 Department of Biomedical, Surgical and Dental Sciences, University of Milan, Via della Commenda 10, 20122 Milan, Italy; 4 Unit of Dental Hygiene, Section of Dentistry, Department of Clinical, Surgical, Diagnostic and Pediatric Sciences, University of Pavia, 27100 Pavia, Italy

**Keywords:** mHealth, mobile applications, oral health, smartphone, dentistry

## Abstract

This scoping review aims to investigate the latest literature concerning the use of smartphone applications (apps) in the prevention, management, and monitoring of oral diseases. Smartphone applications are software programs that are designed to run on smartphones. Nowadays, smartphones are regularly used by people of all ages, and mobile health apps (MHAs) represent an important means of spreading information related to oral health, which is the state of the mouth and teeth, including the gums and other tissues. Several apps have been designed to promote prevention, diagnosis, and therapeutic adherence monitoring. This scoping review considered randomized clinical trials, cross-sectional studies, before–after (pre–post) studies with no control group, and observational studies. Once the inclusion and exclusion criteria had been defined, a preliminary confined search was performed on PubMed and Scopus; key terms from the collected articles were selected to design a search strategy, and then a search of all the included articles’ reference lists was run for further research. Studies were excluded if they did not fulfill the inclusion criteria. The preferred reporting items for scoping reviews (PRISMA-ScR) consensus was followed. The risk of bias was evaluated by providing a qualitative analysis of the clinical studies via the National Heart, Lung, and Blood Institute (NHLBI) Quality Assessment of Controlled Intervention Studies, Observational Cohort Studies, and Cross-Sectional Studies (NHLBI, NIH). A total of 21 studies were included in this review. As it is clear from the studies selected, the literature indicates that MHAs are effective in improving oral hygiene in adolescents and children and reducing the dental plaque index, including in patients undergoing orthodontic treatment. MHAs are also able to reduce the symptoms of patients affected by obstructive sleep apnea–hypopnea syndrome (OSAHS) and improve the swallowing-related quality of life of elderly patients. MHAs are furthermore recommended to decrease dental anxiety among patients, both during dental procedures and the post-operative period. MHAs are useful to spread knowledge about traumatic dental injuries among non-oral health professionals and to monitor dental erosion and awake bruxism. MHAs’ clinical outcomes might have been influenced by the demographic features of the subjects involved. Further studies considering a longer follow-up period and larger samples are needed. In conclusion, MHAs can be considered a useful tool to monitor oral disease and increase patients’ quality of life related to oral health.

## 1. Introduction

The concept of “mobile health” (mHealth) refers to the promotion of healthcare through mobile apps and wireless connections [1].

Nowadays, mobile phones are regularly used by people of all ages and may also be useful in medicine to promote prevention and healthy behaviors, allowing everyone to access reliable information anytime and anywhere [2]. These tools aim to monitor significant factors related to diet, exercise, and therapeutic adherence, which play an important role in several diseases [3,4,5]. The development of mobile apps was meant to promote prevention, diagnosis, disease management, and therapeutic adherence monitoring [6].

In recent years, dentistry has made steps forward as far as research and technological innovation are concerned, leading to significant progress in all the main dental fields [7].

The invention of 3D printing has revolutionized the production of drill guides and implants in oral surgery and of physical models in orthodontics and prosthodontics [8].

Intraoral scanners have become a fundamental part of the digital workflow; digital impressions allow a more precise bite registration, avoiding distortions related to analogue procedures [9].

Nowadays, it is possible to combine 3D printing and intraoral and extraoral scans to design and manufacture customized appliances for patients affected by craniofacial disorders [10].

Some authors have experimented with the ability of AI (artificial intelligence) to diagnose oral diseases, such as head and neck cancer lesions and periodontitis, but also to make therapeutic decisions, for example, to choose between an extractive and a non-extractive treatment for an orthodontic patient [11].

Research about the use of AI in dentistry is currently growing, involving mainly radiological diagnostic imaging [12].

AI can also play a significant role in orthodontics, helping clinicians to make diagnoses, manage orthodontic treatments, assess patients’ compliance, and make patients feel more involved and cared for [13].

The introduction of teledentistry, a combination of telecommunications and dentistry, has represented an interesting innovation to improve oral healthcare [14].

MHAs represent one of the latest trends arousing interest in the literature [7].

MHAs also represent an important means of spreading oral health care information [15]. In the last few years, an increasingly high number of available health-related apps has been evident, reaching 325,000 apps in 2017 [16], with 612 oral health-related apps that could be downloaded from the App Store in 2019 [17]. MHAs provide meaningful information as far as different fields of dentistry are concerned. They motivate patients to maintain good oral hygiene using positive reinforcement to prevent dental caries [18]. These tools could be particularly helpful for orthodontic patients to avoid plaque accumulation, gingival inflammation, and dental caries, which can lengthen treatment times and result in periodontal health worsening [19].

Studies have confirmed that facial scans obtained using mobile applications may be accurate enough for orthodontic assessments [20].

MHAs also help patients to manage and overcome dental anxiety, which is still very common despite technological advances in modern dentistry [21]. Furthermore, some apps have been developed to increase parents’ awareness about oral health in early childhood [22], for example, by teaching their children good eating habits and proper oral health practices [23]. Other MHAs, on the contrary, are addressed to elderly people and provide specific oral exercises that could increase salivary flow rate, reduce xerostomia, and improve swallowing [24]. Moreover, some MHAs have been created to monitor jaw-muscle behaviors, such as awake bruxism [25].

Other scoping reviews in the literature investigated the role of MHAs in dentistry. Vaid et al. analyzed the importance and clinical significance of MHAs in orthodontics [26]. Ben-Omran et al. evaluated the use of MHAs to monitor older adults’ oral health [27].

Considering the significant increase in MHAs related to oral healthcare in the last decade, the present scoping review aims to:

- Analyze the latest literature regarding the prevention, management, and monitoring of oral diseases using MHAs;
- Evaluate the clinical outcomes of MHAs in different fields of dentistry among people of different ages;
- Define future perspectives for the research on MHAs.

## 2. Materials and Methods

### 2.1. Focused Questions

Do mobile health apps (MHAs) provide clinical advantages in dentistry? Are they useful to improve oral healthcare?

### 2.2. Eligibility Criteria

The inclusion criteria adopted in this review are set out below:

(I) Study model: interventional studies, observational studies, cohort studies, case series/case reports studies;
(II) Participants: adolescents, adolescents with fixed orthodontic appliances, mothers of small children, oral practitioners, patients with severe apnea–hypopnea sleep obstructive syndrome, elderly patients affected by systemic disease or having relied on oral health care professionals, patients with fixed orthodontic appliances, dental practice patients, children, adult patients, healthy dental students;
(III) Interventions: use of MHAs related to oral healthcare;
(IV) Outcome: clinical results of the use of MHAs related to oral healthcare.

Only studies fulfilling all the inclusion criteria were taken into consideration.

The exclusion criteria adopted are set out below:

(I) Abstracts of articles written and published in languages different from English;
(II) Duplicate studies;
(III) Non-relevant studies (full-text articles whose purpose was not appropriate to answer the focused questions; analysis of different supplementary treatments; full-text content not corresponding to the abstract);
(IV) No Ethics Committee approval was provided;
(V) Narrative reviews, systematic reviews, or systematic and meta-analysis reviews.

### 2.3. Search Strategy

In accordance with the *Journal of Biomedical Informatics* (JBI) methodology for scoping reviews, a three-step searching process has been followed: (I) preliminary confined search on PubMed (MEDLINE) and Scopus; (II) selection of key terms from collected articles to design a search strategy; (III) search of all included articles’ reference lists for further research [28].

Furthermore, the person-centered care (PCC) model was followed; it is focused on the following three aspects: population (patients and dentists using MHAs related to oral healthcare), concept (using MHAs related to oral healthcare), and context (in this regard, this review does not provide restrictions to any specific cultural issue or setting). Abstracts of studies concerning clinical outcomes of MHAs related to oral healthcare were analyzed. The present scoping review was performed following the preferred reporting items for scoping reviews (PRISMA-ScR) consensus (Table S1 Supplementary Material) [29].

### 2.4. Research

The medical subject heading (MeSH) terms used were mobile applications, mHealth, oral health, and smartphone. Online research was conducted on PubMed (MEDLINE) and Scopus databases. The last search was performed on 31 May 2023. The articles selected were published between 2017 and 2022. Data were extracted from the articles selected between February 2023 and May 2023. The search was performed by three calibrated reviewers (M.G.N., M.P. and M.P.). Disagreements and discrepancies were resolved by consensus, and three other reviewers were involved (A.S., S.G. and P.Z.). All the previously collected articles’ titles and abstracts were carefully analyzed, excluding non-relevant studies. All relevant articles were reviewed by analyzing their full texts, documenting the findings, and detecting any similar studies that followed the inclusion criteria adopted.

The present protocol has been registered within the Open Science Framework platform (Registration DOI-10.17605/OSF.IO/A9CX2).

The elaborated strategies designed for each online database are exhibited in Table S2 (Supplementary Material).

### 2.5. Quality Assessment of Included Studies

In the present review, the risk of bias was evaluated by providing a qualitative analysis of the clinical studies via the National Heart, Lung, and Blood Institute (NHLBI) Quality Assessment of Controlled Intervention Studies, Observational Cohort Studies, and Cross-Sectional Studies (NHLBI, NIH).

## 3. Results

**Figure 1:**
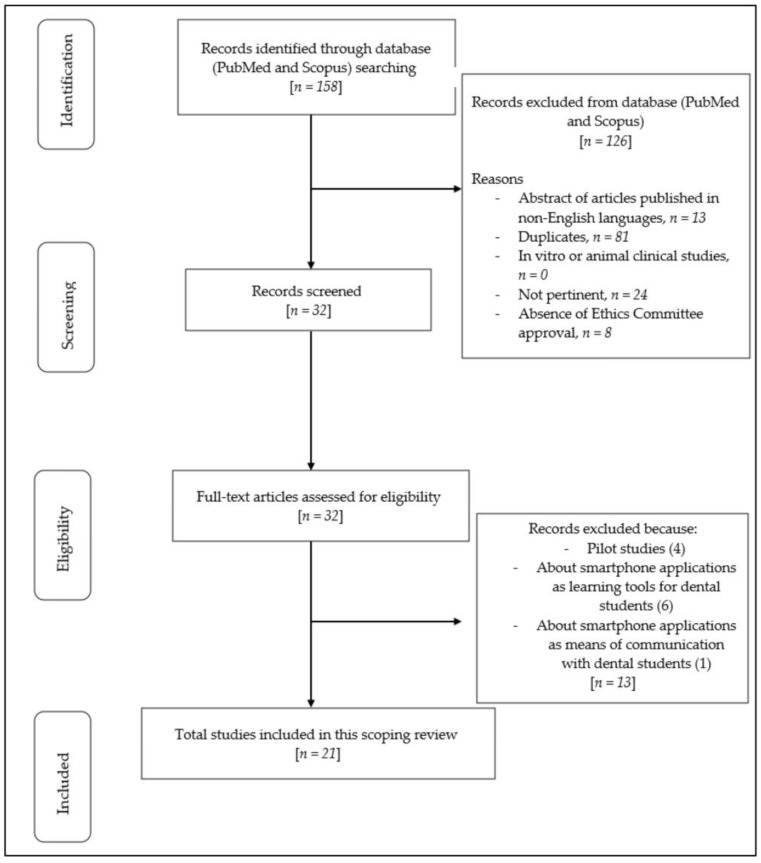
PRISMA-ScR flow diagram.

Based on the MeSH terms, 158 articles were identified in the primary search. Subsequently, 126 articles were discarded (13 abstracts of articles published in languages different from English, 81 duplicates, 0 in vitro or animal clinical studies, 24 not pertinent, and 8 without Ethics Committee approval), and 32 articles were screened based on their titles and abstracts. The remaining 32 full-text articles were assessed for eligibility. Moreover, 11 full-text articles were further discarded as not relevant (4 articles were excluded because they were pilot studies that needed further insights, 6 articles were excluded since they evaluated mobile applications as a learning tool for dental students, and 1 article was excluded since it considered the role of smartphone applications as a means of communication with dental students). The remaining 21 articles were considered relevant and thus included in this review. Figure 1 below describes the flow chart of the review process.

Table S3 (Supplementary Materials) shows the studies excluded from this review and the reasons for exclusion [30,31,32,33,34,35,36,37,38,39,40].

The studies belonged to four categories: randomized controlled clinical trials [41,42,43,44,45,46,47,48,49,50,51], cross-sectional studies [52], before–after (pre–post) studies with no control group [53], and observational studies [54,55,56,57,58,59,60].

### Risk of Bias

The Cochrane Collaboration tool was applied to assess the risk of bias in the articles included in this review (Table 1), using the judging criteria for risk of bias shown in Table S4 (Supplementary Materials). A moderate risk of bias was observed in this review.

The key features of the patients included in the selected studies are set out in Table 2.

Table S5 (Supplementary Materials) explains the evidence of the studies included in this review (study design and aim, methods, results, and conclusions).

Table S6 (Supplementary Materials) illustrates the NHLBI Quality Assessment Tool for Controlled Intervention Studies. The NHLBI Quality Assessment Tool for Cross-Sectional Studies is shown in Table S7. The NHLBI Quality Assessment Tool for before–after (pre–post) studies with no control group is represented in Table S8 (Supplementary Materials). The NHLBI Quality Assessment Tool for Observational Cohort Studies is provided in Table S9 (Supplementary Materials).

## 4. Discussion

In the last few years, mHealth has improved to become a useful tool in modern dentistry. It allows practitioners to collect data about oral healthcare and to constantly monitor and motivate patients. Many MHAs have been developed to spread oral health information among people of all ages. MHAs are meant to educate patients about the importance of proper oral hygiene, which is essential for good oral health [61]. Apps for smartphones and tablets provide patients with reliable information and alert them through push notifications to raise awareness about proper dental behaviors [62]. MHAs could therefore be used to prevent, manage, and monitor oral disease, promoting a more active and appealing involvement of patients in their oral healthcare. As described in the recent literature, several authors tested many MHAs on patients of different ages to assess their effectiveness in preventing oral diseases, spreading positive behaviors, and promoting good oral hygiene.

The studies included in this review showed that MHAs are effective tools to improve the prevention, management, and monitoring of oral disease. Nevertheless, MHAs’ outcomes might differ according to the demographical features of the subjects to whom they are addressed, affecting their effectiveness in some contexts. Furthermore, MHAs’ outcomes should be evaluated over a longer follow-up period and on larger samples.

The present scoping review aims to evaluate the clinical outcomes of MHAs in different branches of dentistry and on patients of different ages, as opposed to other studies published in the literature focusing on single fields and patients of a particular age.

This scoping review aims to detect the clinical outcomes of MHAs related to oral healthcare in the general population.

### 4.1. Oral Hygiene

The role of microbial plaque in the etiology of dental caries and periodontal disease is well-known and documented [63].

Although most of the population performs daily oral hygiene, several studies have reported that most individuals fail to reduce their mouth plaque scores [64].

According to Toniazzo et al., MHAs can represent an effective tool to improve patients’ oral hygiene [65].

Some studies showed that MHAs providing patients with brushing instructions resulted in raising awareness of the importance of proper brushing and improving patients’ brushing techniques [42,44].

MHAs have proved to be effective in reducing mouth plaque scores and, consequently, periodontal inflammation and gingival bleeding [42,44,47]. Alkadhi et al. observed that MHAs providing oral hygiene instructions can the decrease dental plaque index compared to verbal oral hygiene instructions, although further studies with longer follow-up periods are recommended. A short-term follow-up is mentioned as the limitation of the study. [41]

Similarly, Alkilzy et al. found out that MHAs are useful to reduce plaque accumulation; the results might have been influenced by Hawthorne effects, according to whom subjects involved in a study may tend to modify their behaviors. Furthermore, a longer follow-up period should be considered [42].

Kay et al. showed that MHAs are successful in improving brushing outcomes, at least in the short term [44].

It has been observed that adolescents prefer receiving oral health information through smartphone applications and social media [66]. In fact, Marchetti et al. showed that the use of a mobile oral health app leads to an improvement in adolescents’ periodontal health over a long period of time. The study was conducted in a single school, and this could be a limitation, even if subjects were selected to be representative of the study population. Another limitation is the absence of validation of the questionnaire used [47].

### 4.2. Children’s Oral Health

Early childhood caries (ECC) is known to be the most common chronic disease in early childhood [67]. Young children are not typically able to perform proper brushing autonomously, so the supervision of parents plays a fundamental role in their oral hygiene [68]. Parents must teach little children health skills and good eating habits [23]. Morais et al. described MHAs in their integrative review as effective tools for children, combining educational and interactive approaches [69].

It has been shown that the use of MHAs improves mothers’ knowledge and practice about children’s oral health. In particular, MHAs were successful in improving children’s gingival status over a long period of time. The study faced some limitations: some smartphones blocked notifications because of security systems, and subjects without smartphones and preschoolers could not take part in it [50]. Moreover, MHAs represent a modern and particularly successful tool to teach children the correct brushing technique, as reported by the work of Desai et al., in which a significant positive impact on children’s brushing skills was noticed compared to traditional oral hygiene instructions. The limitations of the study are that the sample may not be representative of the study population, a longer follow-up should be taken into consideration, subjects not using a smartphone cannot be involved in the study, and tongue cleaning was not contemplated. Furthermore, the study promoted the modified bass technique, which is difficult to learn for children [43]. A reduced dental plaque index and better hygienic control were observed in children whose mothers used oral health mobile applications. Nevertheless, it would be necessary to analyze MHAs’ outcomes on dental caries over time and to investigate mothers’ oral hygiene to seek a correlation between mothers’ and children’s oral healthcare [51]. Oral health practitioners have shown to be favorable to advising MHAs to little children’s parents. Further studies are recommended, extending the research to all the existing app stores and adopting different scales to evaluate clinical outcomes in children’s oral hygiene [52].

### 4.3. Severe Apnea–Hypopnea Sleep Obstructive Syndrome

Obstructive sleep apnea–hypopnea syndrome (OSAHS) causes impaired sensorimotor deficits in the upper airway muscles [70]. Myofunctional therapy with daily exercises is one of the most novel treatments designed to reinforce the oropharyngeal muscles to avoid the collapse of the upper airways [71]. According to the recent literature, MHAs provide healthy sleep habits and raise enthusiasm among patients with OSAHS, although further studies are needed to achieve major accuracy and reliability in these apps [72].

Patients can learn oropharyngeal exercises using MHAs. It has been shown that oropharyngeal exercises performed with the support of mobile applications reduce OSAHS gravity and symptoms. Future studies involving a large number of participants are encouraged to support this evidence. [48].

### 4.4. Compliance and Duration of Treatment of Orthodontic Patients

In recent years, research has led to significant advances in fixed orthodontics, improving bonding techniques with high-performance and innovative materials [73]. It is well documented that fixed orthodontic appliances make it more difficult to maintain good oral hygiene [74]. As a result, patients with fixed orthodontic appliances might undergo plaque accumulation, which can lead to the development of white spot lesions or even dental caries [75]. The duration of orthodontic treatment might be influenced by behavioral factors such as missed appointments, an unplanned debonding of brackets, and bad oral hygiene, which are signs of poor patient compliance [76].

According to a systematic review, professionals should recommend MHAs since they can be effective in reducing the duration of orthodontic treatment and the intensity of self-reported pain among orthodontic patients [77]. Furthermore, MHAs can also remind patients about elastic and mobile device wear, promote better oral hygiene, and result in earlier treatment outcomes [78].

However, it has been observed that a very low to moderate level of evidence supports the effects of MHAs in improving orthodontic patients’ behaviors [79].

It has been shown that MHAs providing oral hygiene instructions and timely reminders through push notifications improve the oral hygiene of patients with fixed orthodontic appliances, leading to reduced plaque indices and gingival inflammation levels [41,49].

According to Li et al., MHAs are effective in reducing orthodontic treatment duration by improving patients’ compliance and decreasing bracket bond failure. It would be necessary to perform the study on larger samples, involving complex orthodontic cases and adopting a longer follow-up period [46].

### 4.5. Oral Care and Swallowing-Related Quality of Living in Elderly Age

Many elderly people experience xerostomia, swallowing alteration, reduced tongue pressure, and functional impairment of the tongue, mouth, and lips [80], which can interfere with proper food intake and digestion and, consequently, a good quality of life [81]. Recently, several MHAs addressed to elderly people have been devised to improve their health and related quality of life [82]. In particular, some MHAs have been designed to teach elderly people to perform oral exercises and intraoral and extraoral massages to improve their oral health. As a result, Ki et al. described positive effects on elderly people’s oral care: tongue pressure increase, oral dryness reduction, basal salivary flow rate increase, and swallowing-related quality of living improvement. Further studies adopting a longer follow-up period and a larger number of participants are required [45].

### 4.6. Dental Anxiety

Despite the impressive technological innovation of modern dentistry in recent years, many people still suffer from dental anxiety [21]. Due to this condition, anxious patients tend to postpone or even avoid dental treatments, with consequent negative outcomes for their oral health and related quality of life [83]. It has been proven that mHealth may be useful to overcome this issue; mobile applications can produce large effects in reducing dental anxiety compared to other non-pharmacological methods [84].

Huang et al. reported that MHAs allow dentists to perform a teleconsultancy assessing both the physical and psychological patient status. Using this effective tool, the oral health practitioner can follow the patient before the dental procedures, helping to manage dental anxiety up to the post-operative period and addressing possible complications. It should be considered that MHAs’ impact on dental anxiety might be influenced by some aspects, such as sex, age, and possible anxiety disorders, which were not homogeneously represented in the study population [53].

### 4.7. Traumatic Dental Injuries

Dental trauma often occurs in children and adolescents; a proper diagnosis and timely treatment are necessary to allow a favorable long-term prognosis [85]. In their review, van Mechelen et al. included 18 MHAs, among which only 1 app recommended the use of mouth guards to prevent dental injuries, while none of them suggested how to manage them [86]. Parents have to be able to properly manage dental trauma. Iskander et al. compared the effectiveness of MHAs and posters to deliver dental trauma information to parents and showed that both these tools were effective [87]. It is also extremely important to spread knowledge about the management of traumatic dental injuries among non-oral health professionals such as teachers, gym instructors, etc., for whom MHAs have been designed. Zaror et al. validated an MHA regarding dental injury identification and related epidemiologic information collection. The next step is to test this MHA in real cases of trauma in different settings (e.g., schools and gyms) to evaluate its usability in stressful conditions [61].

### 4.8. Dental Erosion

The prevalence of dental erosion is increasing, mainly among young people [88]. This process has a multifactorial etiology and, if not correctly diagnosed and treated, can lead to esthetic and functional problems [89]. MHAs have been designed to improve the management of dental erosions. These tools are addressed both to oral practitioners and patients, promoting a stronger relationship between professionals and patients. Butera et al. demonstrated that oral practitioners can use this type of app to monitor the status of patients’ dental erosion over time, detecting possible progressions in the erosive process, while patients can receive personalized oral hygiene instructions from oral practitioners to avoid further deterioration. Preliminary clinical results have been encouraging, and important enhancements to these apps are expected in the future [54,90].

### 4.9. Awake Bruxism

Bruxism has been described as a jaw-muscle behavior in otherwise healthy individuals [91]. Several studies recommended the use of ecological momentary assessment (EMA) principles to study awake bruxism [25]. A smartphone application has been developed for the EMA of awake bruxism. By sending push notifications, it alerts patients and collects data about their jaw-muscle condition in real time. A good compliance rate has been detected, encouraging further adoption of this tool both for clinical management and research [56]. Câmara-Souza et al. managed to evaluate awake bruxism frequency in college preparatory students in correlation to psychological factors thanks to MHAs. The authors did not find any difference as far as compliance is concerned between workdays and weekends but noticed that some subjects showed a lack of compliance, probably due to the impossibility of using their smartphones during the day and, consequently, of reacting to the alerts. Therefore, it would be necessary to rely on other technological devices, such as smartwatches [55].

According to the studies included, MHAs are an effective tool to gather data about awake bruxism that can be used both for clinical activity and research. In fact, these data enable patients to become aware of their habits and monitor their changes over time, promoting a deeper knowledge of this condition [39,58].

It is desirable to carry out further studies involving MHAs to detect the frequency of AB in healthy subjects and in subjects with conditions such as orofacial pain, sleep disorders, and psychosocial impairment to better analyze the correlation with this phenomenon [57,59].

### 4.10. Limitations and Future Perspectives

This scoping review has some limitations: The results might have been influenced by demographical factors, such as age and geographic localization. Eight studies were excluded because of the absence of Ethics Committee approval. MHA development involves high costs both for professionals and patients; therefore, it might be difficult and expensive to develop and maintain high-quality applications. A further challenge is undoubtedly meeting the requirements of different populations with different levels of oral knowledge and healthcare. The approach should be adapted to the population groups; for example, MHAs for children should be provided with gamification to promote their use.

Furthermore, some MHAs need to be improved as far as accuracy and reliability are concerned to be effective.

It is desirable that future studies evaluate long-term results to confirm MHAs’ effectiveness and use them as part of daily clinical activities. Furthermore, studies involving larger samples are needed. MHAs’ success in different populations and settings and for longer periods should be investigated. It would be necessary to design more user-friendly and engaging MHAs to entice consumers to use them. MHAs should be advertised by dental practitioners to make patients aware of their existence and benefits. The ethical and legal implications of the use of MHAs should be carefully considered. It would also be interesting to combine MHAs with AI, which is an arousing and current research topic.

## 5. Conclusions

Several MHAs have been recently designed to promote the prevention, diagnosis, and therapeutic adherence monitoring of oral disease. MHAs seem to be effective in improving adolescents’ and children’s oral hygiene, including patients undergoing orthodontic treatment; promoting proper oral behaviors; raising awareness about dental injuries; reducing dental anxiety; monitoring oral disease and parafunctions; and increasing patients’ oral health-related quality of life. These outcomes should encourage researchers to enhance existing MHAs and design new ones, improving some features, such as user-friendliness and appeal. Patients should be informed about the positive clinical results of MHAs to encourage them to trust these innovative tools. Further studies are required to evaluate the results in the long term and to assess their possible use as part of daily clinical activities. Most studies included in this review provided a short follow-up period; it would be interesting to observe MHAs’ clinical outcomes in the long term while also investigating patients’ compliance and interest over time.

## Figures and Tables

**Table 1:** Risk of bias of the studies included in this review: the green symbol represents a low risk of bias, while the yellow symbol represents a high risk of bias.

	Random Sequence Generation	Allocation Concealment	Blinding	Incomplete Outcome Data	Selective Reporting
Alkadhi et al., 2017 [41]	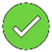	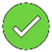	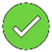	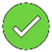	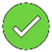
Alkilzy et al., 2019 [42]	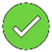	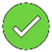	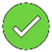	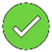	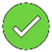
Butera et al., 2022 [54]	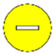	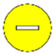	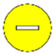	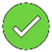	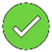
Câmara-Souza et al., 2020 [55]	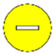	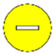	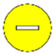	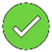	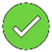
Colonna et al., 2019 [56]	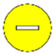	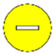	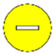	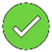	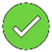
Desai et al., 2021 [43]	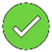	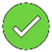	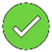	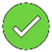	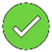
Huang et al., 2022 [53]	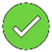	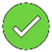	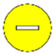	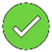	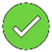
Kanoute et al., 2022 [52]	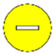	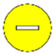	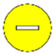	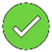	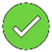
Kay et al., 2019 [44]	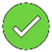	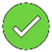	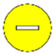	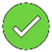	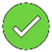
Ki et al., 2021 [45]	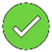	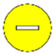	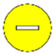	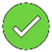	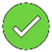
Li et al., 2016 [46]	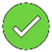	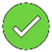	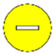	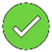	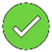
Marchetti et al., 2018 [47]	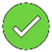	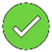	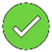	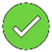	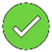
Nykänen et al., 2023 [57]	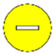	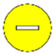	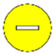	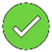	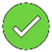
O’Connor-Reina et al., 2020 [48]	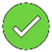	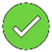	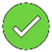	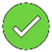	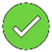
Osiewicz et al., 2019 [58]	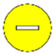	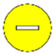	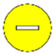	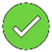	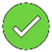
Scheerman et al., 2020 [49]	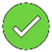	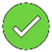	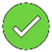	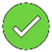	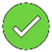
Shirmohammadi et al., 2022 [50]	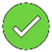	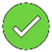	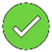	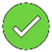	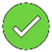
Stanisic et al., 2023 [39]	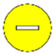	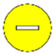	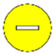	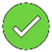	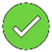
Zani et al., 2019 [59]	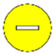	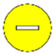	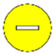	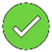	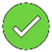
Zaror et al., 2019 [60]	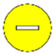	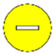	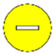	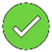	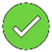
Zolfaghari et al., 2021 [51]	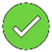	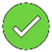	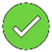	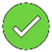	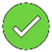

**Table 2:** Baseline characteristics of patients included in the selected studies.

References (Authors, Year of Publication, and Study Design)	No. of Participants Women (W) Men (M)	Age (Years), Mean (SD or Range)	Inclusion and Exclusion Criteria	Clinical Outcome
Alkadhi et al., 2017; RCT [41]	Group 1: 22 W: 11 M: 11 Group 2: 22 W: 14 M: 8	Group 1: 16.6 ± 3.2 Group 2: 17.2 ± 5.2	Inclusion criteria: - Patients with orthodontic fixed appliance treatment; - Patients aged 12 years old and above; - Owning mobile phones; - Patients willing to comply with given oral hygiene instructions. Exclusion criteria: - Not having mental or physical disabilities.	Oral hygiene improvement in patients with fixed orthodontic appliances.
Alkilzy et al., 2019; RCT [42]	Group 1: 26 Group 2: 23 W: 27 M: 22	5.1 ± 0.62	Inclusion criteria: - Aged 5 to 6; - Almost complete deciduous dentition; - Owning a smartphone with an iOS/Android operating system. Exclusion criteria: - Severe general conditions; - Orthodontic appliances; - Motor restrictions.	Improvement in toothbrushing.
Butera et al., 2022; OS [54]	Group 1: 1839 W: N.R. M: N.R. Group 2: 3894 W: 2002 M: 1892	Group 1: N.R. Group 2: 36.72 ± 14.52	Inclusion criteria: N.R. Excluded criteria: N.R.	Dental erosion evaluation.
Câmara-Souza et al., 2020 [55]	69 W: 50 M: 19	18.6 ± 1.5	Inclusion criteria: - Being regularly enrolled in the college preparatory exam course; - Having a cell phone compatible with the dedicated EMA application; - Being dentate; - Having general good health. Exclusion criteria: - Any ongoing medical, psychological, or pharmacological treatment; - Report of TMD or any other orofacial pain conditions; - History of any therapy for AB or TMD in the previous 12 months.	Correlation of AB frequency with levels of anxiety, depression, stress, and OHRQoL in college preparatory students.
Colonna et al., 2019; OS [56]	60 W: 35 M: 25	24.2 ± 4.1	Inclusion criteria: - Healthy dental students attending the last 3 years of School of Dentistry at the University of Ferrara. Exclusion criteria: - Presence of temporomandibular disorder (TMD) pain and/or any documented neurological, psychiatric, sleep, or systemic (e.g., rheumatologic, hormonal) diseases.	Awake bruxism evaluation.
Desai et al., 2021; RCT [43]	Group 1: 82 Group 2: 83 Group 3: 82 W: 121 M: 126	4.98 ± 0.84	Inclusion criteria: - Aged 4 to 6; - Present on the day of examination; - Owning a smartphone. Exclusion criteria: N.R.	Improvement in children’s oral hygiene.
Huang et al., 2022; BAS [53]	Group 1: 180 W: 104 M: 76 Group 2: 20 W: 9 M: 11	Group 1: 3 to 74 Group 2: N.R.	Inclusion criteria: - Ability to access the internet via cellular data or Wi-Fi with smartphones, either independently or with the help of relatives. Exclusion criteria: - Inability to use the smartphone to complete the questionnaire.	Dental anxiety evaluation.
Kanoute et al., 2022; CSS [52]	10 W: N.R. M: N.R.	N.R.	Inclusion criteria: - Being an OHP and/or being in or having been in a dental practice in SSA. Exclusion criteria were: - Not owning a smartphone; - Inability to download applications from the iOS (App Store) or Android (Google Play Store) stores; - Lack of experience in using mobile applications; - Having hearing, visual, or motor disabilities.	Children’s oral hygiene evaluation.
Kay et al., 2019; RCT [44]	Group 1: 53 W: 34 M: 19 Group 2: 51 W: 27 M: 24	Group 1: 36.6 ± N.R. Group 2: 39.1 ± N.R.	Inclusion criteria: - Dental practice patients. Exclusion criteria: N.R.	Improvement in oral hygiene.
Ki et al., 2021; RCT [45]	Group 1: 20 W: 10 M: 10 Group 2: 20 W: 13 M: 7	≥65	Inclusion criteria: - Comprehension of the design and the aim of the study; - Will to participate; - Capability to communicate in the absence of linguistic, auditory, or visual disabilities; - Normal cognitive capacity. Exclusion criteria: - Skipping at least two sessions of the program; - Oral health behaviors’ rate of practice inferior to 80%; - A history of systemic disease that could affect oral health (drugs affecting saliva secretion, Sjögren syndrome, oral cancer, and stroke).	Improvement in oral health and swallowing-related quality of life.
Li et al., 2016; RCT [46]	Group 1: 112 W: 79 M: 33 Group 2: 112 W: 77 M: 35	Group 1: 17.6 ± 0.8 Group 2: 18.7 ± 1.0	Inclusion criteria: - Adolescents or adults admitted for orthodontic treatment; - Orthodontic patients with fixed appliances and single-phase treatments. Exclusion criteria: - Unable to read Chinese; - Impossibility of using a smartphone and installing the WeChat app; - Preference for lingual or invisible bracketless technique; - Planned for a multiphase treatment, like combined orthodontic–orthognathic treatment; - Too complicated to be finished within 3 years; - Chance to migrate to another city within the predicted treatment period.	Orthodontic patients’ compliance and duration of treatment evaluation.
Marchetti et al., 2018; RCT [47]	291 W: 159 M: 132	16.1 ± 1.21	Inclusion criteria: - Adolescents of both sexes; - Aged 14–19 years; - Enrolled in a technical high school in the city of Curitiba, Parana, Brazil. Exclusion criteria: - Adolescents with some physical or mental condition that made interventions impossible; - Adolescents using fixed orthodontic devices at the time of clinical examination.	Adolescents’ periodontal health improvement.
Nykänen et al., 2023 [57]	Group 1: 68 W: 60 M: 8 Group 2: 47 W: 41M: 6	Group 1: 45.7 ± 10.6 Group 2: 43.5 ± 9.8	Inclusion criteria: - Presenting a poor response to TMD treatment provided according to the Finnish National Guidelines for TMD management. Exclusion criteria: - Being under 18 years of age.	AB’s prevalence evaluation.
O’Connor-Reina et al., 2020; RCT [48]	Group 1: 18 W: 4 M: 14 Group 2: 10 W: 2 M: 8	Group 1: 59.17 (53.7–64.6) Group 2: 63.9 (56.4–71.38)	Inclusion criteria: - Aged 18 to 75; - Recently diagnosed with severe sleep apnea but with no previous experience with this pathology; - Provision of informed written consent. Exclusion criteria: - BMI > 40 kg/m^2^; - Inability to complete the questionnaires; - Severe drug or alcohol abuse; - Use of hypnotic medication; - Uncontrolled coronary disease; - Decompensated heart failure; - History of stroke; - Systemic disease associated with an inflammatory-related entity (e.g., arthritis, sarcoidosis, vasculitis, lupus); - Neuromuscular disease (e.g., Duchenne muscular dystrophy); - Craniofacial deformity; - Active oncology; - Any previous use of MT treatment or other treatments for sleep apnea that could affect the study results (e.g., surgery, MAD, or CPAP).	OSAHS severity and symptoms.
Osiewicz et al., 2019 [58]	N.R.	N.R.	N.R.	Bruxism evaluation.
Scheerman et al., 2020; RCT [49]	Group 1: 67 W: 41 M: 26 Group 2: 65 W: 32 M: 33	Group 1: 13.2 ± 1.01 Group 2: 13.5 ± 0.97	Inclusion criteria: - Adolescents with fixed orthodontic appliances visiting orthodontic clinics in Alkmaar and Leiden. Exclusion criteria: N.R.	Oral hygiene improvement in patients with fixed orthodontic appliances.
Shirmohammadi et al., 2022; RCT [50]	Group 1: 45 Group 2: 45 W: 90 M: 0	35.6 ± 5.0	Inclusion criteria: - Owning a smartphone; - Mother of children aged 2 to 6. Exclusion criteria: - Unwillingness to attend the trial; - Mother of children with systemic diseases and health conditions.	Children’s oral health improvement.
Stanisic et al., 2023 [39]	Group 1: 10 W: 6 M: 4 Group 2: 10 W: 7 M: 3	Group 1: aged between 23 and 30 Group 2: aged between 42 and 67	Inclusion criteria: N.R. Exclusion criteria: N.R.	Awake bruxism evaluation.
Zani et al., 2019 [59]	30 W: 21 M: 9	24 ± 3.5	Inclusion criteria: - Being an undergraduate student attending different university courses. Exclusion criteria: - N.R.	Awake bruxism evaluation.
Zaror et al., 2019; OS [60]	182 W: 129 M: 53	36.2 ± 9.3	Inclusion criteria: - Participants from health care centers. Exclusion criteria: N.R.	Traumatic dental injury evaluation.
Zolfaghari et al., 2021; RCT [51]	Group 1: 29 Group 2: 29 W: 58 M: 0	Group 1: 36.5 ± 4.9 Group 2: 36.3 ± 4.5	Inclusion criteria: - Owning a smartphone; - Mother of children not older than 6. Exclusion criteria: N.R.	Children’s oral health improvement.

Legend: W: Women. N.R.: Not reported. BMI: Body Mass Index. MT: Myofunctional therapy. MAD: Mandibular Advancing Device. CPAP: Continuous Positive Airway Pressure. OSAHS: Severe apnea–hypopnea sleep obstructive syndrome. OHP: Oral health professional. SSA: Sub-Saharian Africa. TMD: Temporomandibular disorders. RCT: Randomized clinical trial. CSS: Cross-sectional study. BAS: Before–after study. OS: Observational study. AB: Awake bruxism.

## Data Availability

Data are available upon motivated request to the corresponding authors.

## Supplementary Materials

The following supporting information can be downloaded at https://www.mdpi.com/article/10.3390/dj11100243/s1, Table S1. PRISMA-ScR checklist.; Table S2: Search strategies for electronic databases.; Table S3. Summary table of studies excluded in this scoping review.; Table S4. Criteria for judging risk of bias in the “Risk of bias” assessment tool.; Table S5: Evidence of studies included in this scoping review.; Table S6. NHLBI Quality Assessment of Controlled Intervention Studies.; Table S7. NHLBI Quality Assessment for Cross-Sectional Studies.; Table S8. NHLBI Quality Assessment Tool for before–after (pre–post) studies with no control group; Table S9. NHLBI Quality Assessment Tool for Observational Cohort Studies.
