# Supplementary material for: Smartphone Applications in Dentistry: A Scoping Review
Source: Dent J (Basel). 2023 Oct 20;11(10):243. doi: 10.3390/dj11100243 (PMC10605491; doi:10.3390/dj11100243)
Supplement: Supplementary file 1 [file dentistry-11-00243-s001.zip › dentistry-2581704-supplementary.pdf]

Table S1. (PRISMA-ScR) Checklist.

SECTION	ITEM	PRISMA-ScR CHECKLIST ITEM	REPORTED ON PAGE #
**TITLE**			
Title	1	Identify the report as a scoping review.	1
**ABSTRACT**			
Structured summary	2	Provide a structured summary that includes (as applicable): background, objectives, eligibility criteria, sources of evidence, charting methods, results, and conclusions that relate to the review questions and objectives.	1
**INTRODUCTION**			
Rationale	3	Describe the rationale for the review in the context of what is already known. Explain why the review questions/objectives lend themselves to a scoping review approach.	1-2
Objectives	4	Provide an explicit statement of the questions and objectives being addressed with reference to their key elements (e.g., population or participants, concepts, and context) or other relevant key elements used to conceptualize the review questions and/or objectives.	1-2
**METHODS**			
Protocol and registration	5	Indicate whether a review protocol exists; state if and where it can be accessed (e.g., a Web address); and if available, provide registration information, including the registration number.	2-3
Eligibility criteria	6	Specify characteristics of the sources of evidence used as eligibility criteria (e.g., years considered, language, and publication status), and provide a rationale.	2-3
Information sources*	7	Describe all information sources in the search (e.g., databases with dates of coverage and contact with authors to identify additional sources), as well as the date the most recent search was executed.	2-3
Search	8	Present the full electronic search strategy for at least 1 database, including any limits used, such that it could be repeated.	2-3
Selection of sources of evidence†	9	State the process for selecting sources of evidence (i.e., screening and eligibility) included in the scoping review.	2-3
Data charting process‡	10	Describe the methods of charting data from the included sources of evidence (e.g., calibrated forms or forms that have been tested by the team before their use, and whether data charting was done independently or in duplicate) and any processes for obtaining and confirming data from investigators.	2-3
Data items	11	List and define all variables for which data were sought and any assumptions and simplifications made.	2-3
Critical appraisal of individual sources of evidence§	12	If done, provide a rationale for conducting a critical appraisal of included sources of evidence; describe the methods used and how this information was used in any data synthesis (if appropriate).	2-3
Synthesis of results	13	Describe the methods of handling and summarizing the data that were charted.	2-3
**RESULTS**			
Selection of sources of evidence	14	Give numbers of sources of evidence screened, assessed for eligibility, and included in the review, with reasons for exclusions at each stage, ideally using a flow diagram.	3-4-5-6-7-8-9

SECTION	ITEM	PRISMA-ScR CHECKLIST ITEM	REPORTED ON PAGE #
Characteristics of sources of evidence	15	For each source of evidence, present characteristics for which data were charted and provide the citations.	3-4-5-6-7-8-9
Critical appraisal within sources of evidence	16	If done, present data on critical appraisal of included sources of evidence (see item 12).	3-4-5-6-7-8-9
Results of individual sources of evidence	17	For each included source of evidence, present the relevant data that were charted that relate to the review questions and objectives.	3-4-5-6-7-8-9
Synthesis of results	18	Summarize and/or present the charting results as they relate to the review questions and objectives.	3-4-5-6-7-8-9
**DISCUSSION**			
Summary of evidence	19	Summarize the main results (including an overview of concepts, themes, and types of evidence available), link to the review questions and objectives, and consider the relevance to key groups.	10-11-12-13
Limitations	20	Discuss the limitations of the scoping review process.	10-11-12-13
Conclusions	21	Provide a general interpretation of the results with respect to the review questions and objectives, as well as potential implications and/or next steps.	10-11-12-13
**FUNDING**			
Funding	22	Describe sources of funding for the included sources of evidence, as well as sources of funding for the scoping review. Describe the role of the funders of the scoping review.	13

Table S2: Search strategies for electronic databases.

Database	Search strategy
PubMed (MEDLINE)	#1 "mobile applications" [MESH] OR (Mobile Apps) OR (Smartphone Apps)<br>#2 "mHealth" [MESH] OR (Tele-Referral) OR (Virtual Medicine) OR (eHealth)<br>#3 "Oral health" [MESH]<br>#4 "Smartphone" [MESH] OR (Smart Phone)<br>#5 #1 AND #4 AND #5<br>#6 #1 AND #3 AND #4<br>#7 #2 AND #4 AND #5 AND<br>#8 #2 AND #3 AND #4 AND #5<br>#9 #1 AND #2 AND #3 AND #4

SCOPUS	#1 “mobile applications” [MESH] OR (Mobile Apps) OR (Smartphone Apps)<br><br>#2 “mHealth” [MESH] OR (Tele-Referral) OR (Virtual Medicine) OR (eHealth)<br><br>#3 “Oral health” [MESH]<br><br>#4 “Smartphone” [MESH] OR (Smart Phone)<br><br>#5 #1 AND #4 AND #5<br><br>#6 #1 AND #3 AND #4<br><br>#7 #2 AND #4 AND #5 AND<br><br>#8 #2 AND #3 AND #4 AND #5<br><br>#9 #1 AND #2 AND #33 AND #4

Table S3. Summary table of studies excluded in this scoping review.

Excluded Studies	Exclusion Reasons
Choi et al. 2022 **[1]**	About smartphone applications as learning tools for dental students
Golshah et al. 2020 **[2]**	About smartphone applications as learning tools for dental students
Humm et al. 2020 **[3]**	Pilot study
Khatoon et al. 2015 **[4]**	About smartphone applications as means of communication with dental students
Matos Lamenha-Lins et al. 2022 **[5]**	About smartphone applications as learning tools for dental students
Mergany et al. 2021 **[6]**	About smartphone applications as learning tools for dental students
Moylan et al. 2019 **[7]**	Pilot study
Panchal et al. 2017 **[8]**	Pilot study
Singh et al. 2019 **[9]**	About smartphone applications as learning tools for dental students
Stanisic et al. 2023 **[10]**	Pilot study
Suner et al. 2019 **[11]**	About smartphone applications as learning tools for dental students

Table S4. Criteria for judging risk of bias in the "Risk of bias" assessment tool.

**Random Sequence Generation**	
Criteria for a judgement of 'Low risk' of bias.	The investigators describe a random component in the sequence generation process.
Criteria for the judgement of 'High risk' of bias.	The investigators describe a non-random component in the sequence generation process. Usually, the description would involve some systematic, non-random approach.<br>Other non-random approaches happen much less frequently than the systematic approaches mentioned above and tend to be obvious. They usually involve judgement or some method of non-random categorization of participants.
**Allocation Concealment**	
Criteria for a judgement of 'Low risk' of bias.	Participants and investigators enrolling participants could not foresee assignment because one of the following, or an equivalent method, was used to conceal allocation.
Criteria for the judgement of 'High risk' of bias.	Participants or investigators enrolling participants could possibly foresee assignments and thus introduce selection bias.
**Blinding**	
Criteria for a judgement of 'Low risk' of bias.	Any one of the following:<br>- No blinding or incomplete blinding, but the review authors judge that the outcome is not likely to be influenced by lack of blinding;<br>- Blinding of participants and key study personnel ensured, and unlikely that the blinding could have been broken;<br>- No blinding of outcome assessment, but the review authors judge that the outcome measurement is not likely to be influenced by lack of blinding;<br>- Blinding of outcome assessment ensured, and unlikely that the blinding could have been broken.
Criteria for the judgement of 'High risk' of bias.	Any one of the following:<br>- No blinding or incomplete blinding, and the outcome is likely to be influenced by lack of blinding;<br>- Blinding of key study participants and personnel attempted, but likely that the blinding could have been broken, and the outcome is likely to be influenced by lack of blinding;

	- No blinding of outcome assessment, and the outcome measurement is likely to be influenced by lack of blinding;<br>- Blinding of outcome assessment, but likely that the blinding could have been broken, and the outcome measurement is likely to be influenced by lack of blinding.
**Incomplete Outcome Data**	
Criteria for a judgement of 'Low risk' of bias.	Any one of the following:<br>- No missing outcome data;<br>- Reasons for missing outcome data unlikely to be related to true outcome (for survival data, censoring unlikely to be introducing bias);<br>- Missing outcome data balanced in numbers across intervention groups, with similar reasons for missing data across groups;<br>- For dichotomous outcome data, the proportion of missing outcomes compared with observed event risk not enough to have a clinically relevant impact on the intervention effect estimate;<br>- For continuous outcome data, plausible effect size (difference in means or standardized difference in means) among missing outcomes not enough to have a clinically relevant impact on observed effect size;<br>- Missing data have been imputed using appropriate methods.
Criteria for the judgement of 'High risk' of bias.	Any one of the following:<br>- Reason for missing outcome data likely to be related to true outcome, with either imbalance in numbers or reasons for missing data across intervention groups;<br>- For dichotomous outcome data, the proportion of missing outcomes compared with observed event risk enough to induce clinically relevant bias in intervention effect estimate;<br>- For continuous outcome data, plausible effect size (difference in means or standardized difference in means) among missing outcomes enough to induce clinically relevant bias in observed effect size;<br>- 'As-treated' analysis done with substantial departure of the intervention received from that assigned at randomization;<br>- Potentially inappropriate application of simple imputation.
**Selective Reporting**	

Criteria for a judgement of 'Low risk' of bias.	Any one of the following:<br>- The study protocol is available and all of the study's pre-specified (primary and secondary) outcomes that are of interest in the review have been reported in the pre-specified way;<br>- The study protocol is not available but it is clear that the published reports include all expected outcomes, including those that were pre-specified (convincing text of this nature may be uncommon).
Criteria for the judgement of 'High risk' of bias.	Any one of the following:<br>- Not all of the study's pre-specified primary outcomes have been reported;<br>- One or more primary outcomes is reported using measurements, analysis methods or subsets of the data (e.g., subscales) that were not pre-specified;<br>- One or more reported primary outcomes were not pre-specified (unless clear justification for their reporting is provided, such as an unexpected adverse effect);<br>- One or more outcomes of interest in the review are reported incompletely so that they cannot be entered in a meta-analysis;<br>- The study report fails to include results for a key outcome that would be expected to have been reported for such a study.

Table S5: Evidence of studies included in this scoping review.

Authors and Year of Publication	Study Design and Aim	Methods	Results	Conclusions
Alkadhi et al. 2017 **[12]**	A 4-week randomized controlled clinical trial to investigate the effect of using mobile applications active reminders to improve oral hygiene in comparison to verbal oral hygiene instructions.	Forty-four 12-year-old and older subjects undergoing orthodontic treatment with fixed appliances were randomly assigned to one of two groups using simple randomisation. Group I: subjects received a mobile application that sends active reminders of oral hygiene three times a day (n = 22). Group II: subjects received verbal oral hygiene instructions verbally during their routine orthodontic visits (n = 22). Two primary outcomes were assessed using plaque index (PI) and gingival index (GI) for Ramfjord teeth to evaluate the level of oral hygiene at baseline and after 4 weeks.	Mean differences for PI and GI for group I were reduced from T1 to T2 ($P < 0.05$, $P < 0.05$) but did not significantly change for group II ($P > 0.05$, $P > 0.05$). Both PI and GI significantly reduced for group I compared to group II between T1 and T2 ($P < 0.05$, $P < 0.05$).	PI and GI all significantly decreased after 4 weeks of using active reminders of oral hygiene instructions on mobile application compared to verbal oral hygiene instructions.
Alkilzy et al. 2019 **[13]**	A 12-week randomized controlled clinical trial to investigate the effect of a smartphone app for improving manual toothbrushing via a gravitation sensor.	In this prospective, controlled, single-blinded, randomized clinical trial, 49 children (mean age 5.1 ± 0.6 years, 27 female) were randomly assigned to test (n = 26) and control (n = 23) groups. All children were provided with manual toothbrushes with an	At baseline, there were no significant differences between the test and control group regarding plaque and gingival indices (QHI: 2.36 ± 0.7 and 2.42 ± 0.8; p = 0.94; PBI: 0.42 ± 0.2 and 0.47 ± 0.3; p = 0.59). At the 6- and 12-week recalls, the test group showed statistically significantly better oral health indices than the	The results highlight the enormous possibilities of a toothbrushing application via the smartphone, at least for medium-term oral hygiene improvement in preschool children and even after excluding the app. The long-term effect should also be investigated to exclude the expected novelty effect.

		integrated gravitation sensor, and they received oral health instructions. Only the children of the test group got an additional smartphone app to visualize and reward proper brushing in form and time. At baseline and recalls after 6 and 12 weeks, plaque and gingival indices (QHI, PBI) were recorded for analysis between the two groups.	controls (6- week recall, QHI: 0.8 ±0.5 and 1.88 ± 0.9; $p < 0.001$; PBI: 0.08 ± 0.1 and 0.26 ± 0.2; $p < 0.001$; 12-week recall, QHI: 0.44 ± 0.5 and 1.49 ± 0.7; $p < 0.001$; PBI: 0.05 ± 0.18 and 0.21 ± 0.1; $p < 0.001$).	
Butera et al. 2022 **[14]**	A 3-year observational study to conduct an initial evaluation on the use of the Intact-Tooth application, which has been available since September 2019.	Photos of 3894 patients with dental erosion were uploaded, through which the degree of susceptibility and the BEWE (basic erosive wear examination index) index could be assessed.	99.72% had a susceptibility grade of 0 to 8, while 0.28% had a medium-high susceptibility grade; this result is related to the age and sex of the patients.	The management of patients through the help of the application could promote the diagnosis and treatment of enamel diseases and encourage the self-learning of the learning machine, thanks to the number of clinical cases uploaded.

Câmara-Souza et al. 2020 [**15**]	A 1-week cross-sectional study to assess the frequency of reported masticatory muscles activity during wakefulness (i.e., awake bruxism [AB]), levels of anxiety, depression, stress, and the oral health-related quality of life (OHRQoL) in college preparatory students.	Sixty-nine college preparatory students participated in the study. AB was evaluated by the Oral Behaviors Checklist (OBC) and a smartphone-based ecological momentary assessment (EMA; [Bruxapp®]). Anxiety and depression were measured by the Hospital Anxiety and Depression Scale, stress was evaluated by the Perceived Stress Scale, and OHRQoL was obtained by The Oral Health Impact Profile-14. Data were analyzed by Pearson's correlation coefficient ($\alpha = 0.05$).	The average EMA-reported frequency of AB behaviors was 38.4%. Significant correlations were found between AB and the OBC, anxiety, depression, stress, and OHRQoL ($p < 0.001$).	College preparatory students demonstrated moderate frequency of AB, which was significantly correlated with psychosocial factors

Colonna et al. 2019 **[16]**	A 1-week observational study to assess the compliance with the use of a smartphone-based ecological momentary assessment (EMA) strategy to collect real time data on awake bruxism (AB) over 1 week in a sample of healthy young adults.	Sixty (N = 60) healthy young adults (mean age 24.2 ± 4.1 years) used a dedicated smartphone application that sent 20 alerts at random times throughout the day. Upon alert receipt, the subjects had to report in real time their condition among five possible options: relaxed jaw muscles, teeth contact, teeth clenching, teeth grinding, and mandible bracing. Compliance rate with the app was assessed at the individual and group level in terms of percentage of answered alerts as well as number of days that were needed to reach the targeted observation period of 7 days with a compliance of at least 60%.	The mean compliance recorded with the smartphone application was 67.8% of the total alerts. On average, 9.8 ± 3.2 days (range 7–19) have been necessary to achieve the targeted goal of 7 days with a minimum of 60% alerts/day. No gender differences were detected in any compliance data. Response rate was not different during weekdays or weekends.	This investigation is the first attempt to assess individual compliance with EMA for reporting awake bruxism. Results suggest that a smartphone-based strategy can have interesting potential. The compliance rate reported in this study will serve as a comparison standpoint for future investigations.

Desai et al. 2021 **[17]**	A 1-month randomized controlled clinical trial to investigate the impact of “Brush Up” - a mobile application, on oral hygiene behaviours of 4-6-year-old children in Bangalore city.	In this experimental study, 247 children aged 4-6, were randomly divided into three groups. Considering “Brush Up” is a mobile application, parents of the children in Group 1 (n=82) downloaded the application on their smartphones. Children in Group 2 (n=83) and Group 3 (n=82) received tooth brushing instructions by an educative video and manual demonstration, respectively. Effectiveness of tooth brushing was assessed with plaque scores, which were recorded for all the groups at baseline and one month using Visible Biofilm Index.	Wilcoxon signed rank test showed a significant improvement in the tooth brushing behaviour for the Brush Up group, which was indicated by a lower plaque score after a follow-up of one month. Kruskal-Wallis test followed by *post-hoc* test showed that the mean ranks of plaque scores of Brush Up group are consistently lower than those of video demonstration group and manual demonstration group.	The lower plaque score in subsequent follow-up in Brush Up group suggests that using a smart system can enhance learning a correct tooth brushing method in young children and can also help in implementing the required reinforcement and motivation to brush and aid in better plaque control.

Huang et al. 2022 **[18]**	A 12-month before-after (pre-post) study with no control group to verify whether WeChat Applet for dental anxiety (WADA)could play a beneficial role before and after a dental procedure and facilitate management of high-risk patients during the COVID-19 pandemic.	During the 12-month survey period (August 2020 to July 2021), a total of 180 patients aged 3–74 years from eight different cities (n = 180 at the end of treatment, n = 25 for the System Usability Scale (SUS) and follow-up interview) and 20 medical staff from eight different cities (n =20 for follow-up interview) were evaluated by WADA. At the end of the survey period, the results of the interviews were analyzed thematically.	WADA assessment results from 180 patients and follow-up interview results from 45 participants were analyzed. In this study with a male to female ratio of 2:3, 75% were found to be suffering from dental anxiety, 86% were found with postoperative complications, and 11 cases were found to have contraindications to surgery. The total SUS score for WADA is 72.25 above the mean score, proving that WADA is a relevant and useful tool before and after dental treatment. Based on the results of the interviews, the following themes were identified: patient satisfaction; dentists' effectiveness; multi-center data integration; and increase its frequency of usage.	The WADA was developed for dental procedures and is effective for reducing treatment risks, improving patients' satisfaction and dentists' convenience, especially in terms of facilitating management of high-risk patient during the COVID-19 pandemic.
Kanoute et al. 2022 **[19]**	A cross-sectional study to assess the quality of mobile applications linked to oral hygiene for children currently featured on the iOS and Android stores in sub-Saharan Africa.	Ten oral health professionals (OHP) used the French Mobile	The highest MARS-F scores for overall quality were reported	Thus, OHP rated positively the quality of the majority of mobile

		App Rating Scale (MARS-F) to rate 15 selected applications.	for *Bonne nuit Caillou* (3.89 ± 0.74), *Mon Raccoon* (3.63 ± 0.95), and Chomper Chums (3.54 ± 0.54) while the lowest MARS-F scores for overall quality were achieved by Brushing time (2.31 ± 0.61), *De belles dents* (2.55 ± 0.55) and Brushing Hero (2.77 ± 0.53). The subjective quality scores ranged from 1.50 ± 0.68 for Brushing time to 3.25 ± 0.97 for *Bonne nuit Caillou*. Specificity scores ranged from 1.95 ± 0.88 (Brushing time) to 3.75 ± 0.84 (*Bonne nuit Caillou*).	applications linked to oral hygiene for children, their effect on users' knowledge, attitudes, and intentions to change, and the probability of effective oral hygiene behavior modification. They stated that they would recommend their use to their patients who need them. However, studies analyzing the change in oral hygiene behavior of children using these apps need to be conducted.
Kay et al. 2019 **[20]**	A 4-week randomized controlled clinical trial to test the effectiveness and acceptability of a smartphone application used in conjunction with a movement sensor toothbrushing attachment for promoting plaque control.	One hundred and eight dental practice patients were recruited to the study from two general dental practices. Participants were randomised to test and control groups, and both groups offered oral hygiene instruction according to a single protocol. Test participants were given	Full mouth plaque scores declined from 40.1 to 11.7, a reduction of 70% in the test group compared to a reduction from 29.1 to 20.5 (30%) in the control group. The device was found to be very well accepted. Participants were conscious of improving their brushing and	Providing immediate day-to-day feedback to dental patients about their brushing results in dramatic improvements in oral hygiene and highly significant reductions in plaque levels, in at least the short-term; beyond that seen in previous toothbrushing

			the smartphone device and toothbrush attachment. Control patients were not. After two and four weeks, full mouth plaque scores of the mouths of both test and control participants were measured. A comprehensive questionnaire administered to the test group assessed participants' views about the acceptability of the smartphone device and application.	improving their knowledge of how to brush well. They also reported enjoyment and fun being derived from use of the device and found it simple to use.	interventions with adult patients-
Ki et al. 2021 **[21]**	A 6-week randomized controlled clinical trial to investigate the effect of oral health education using a mobile app (OHEMA) on the oral health and swallowing-related quality of life (SWAL-QoL) of the elderly population in a community-based integrated care project (CICP).	Forty elderly individuals in the CICP were randomized into intervention and control groups. OHEMA provided information on customized oral health care management, oral exercises, and intraoral and extraoral massage methods for 50 min/session, once a week, for 6 weeks. Pre- and post-intervention surveys assessed the unstimulated salivary flow rate, subjective oral dryness, tongue pressure, and SWAL-QoL, which were analyzed using ANCOVA and repeated measures ANOVA.	In the intervention group, tongue pressure increased significantly from pre- (17.75) to post-intervention (27.24) ($p < 0.001$), and subjective oral dryness decreased from pre- (30.75) to post-intervention (18.50). The	OHEMA appears to be a useful tool for oral health education for the elderly as it improved the SWAL-QoL, with increased tongue pressure and reduced oral dryness.	

		unstimulated salivary flow rate had a higher mean score in the intervention group (7.19) than in the control group (5.04) ($p < 0.001$). The SWAL-QoL significantly improved from pre- (152.10) to post-intervention (171.50) in the intervention group ($p < 0.001$) but did not change significantly in the control group ($p > 0.05$).		
Li et al. 2016 **[22]**	A 33-month randomized controlled clinical trial to evaluate the effect of intervention using a messaging app (WeChat) on compliance and duration of treatment in adult or adolescent patients with fixed orthodontic appliances in Chengdu, China.	A randomized controlled trial was performed in a dental hospital and a clinic from August 2012 to May 2015. Orthodontic patients were included at the beginning of treatment. Patients with multiphase treatment or braceless technique were excluded. Participants were	One hundred twelve patients in each group participated and completed the trial. DOT in WeChat group were 7.3 weeks shorter (P = 0.007). There were less failed attendance (3.1 vs. 10.9 %, P < 0.001), late attendance (20.1 vs. 29.9 %, P < 0.001), and bracket bond failure (11.8 vs.	The intervention with WeChat is effective in reducing the treatment duration and bracket bond failure and improving the attendance in orthodontic patients. DOT can be reduced by improving patient's compliance. The messaging app is useful

		randomized to WeChat group (received regular reminders and educational messages) or control group (received conventional management) and were followed up until the treatment was completed. Primary outcome measure was DOT. Others were late and failed attendance, bracket bond failure, and oral hygiene condition.	16.1 %, P < 0.001) in WeChat group than control. There was no difference in orthodontic plaque index nor modified gingivitis index between the two groups before and after treatment. Number of failed attendances was identified as an independent factor affecting DOT (P = 0.004; HR = 0.89, 95 % CI 0.84 to 0.95).	for outpatient education and management.
Marchetti et al. 2018 **[23]**	A 30-day randomized controlled clinical trial to evaluate the influence of an App associated with conventional educational methods in adolescents' oral health.	Randomized controlled trial including 291 participants (mean age = 16.1 years) in baseline. The study consisted of four phases. Interventions were evaluated through the knowledge score (KS) and oral indexes (OHI-S/GBI). KS was obtained through five affirmations about periodontal diseases applied in different moments (pre-test, post-test, and follow-up test). Phase I included pre-test and oral	Marchetti et al. 2018 **[Marchetti et al. 2018]**	A 30-day randomized controlled clinical trial to evaluate the influence of an App associated with conventional educational methods in adolescents' oral health.

		clinical examination. Sample was randomly divided into two groups: oral (OG) and video orientation (VG) and post-test (phase II). Phase III characterized the formation of groups: OG + App/OG without App/VG + App/VG without App. App consisted of reinforcement messages which was sent during 30 days. Phase IV comprised follow-up test and clinical evaluation.		
Nykänen et al. 2023 [**24**]	A 1-week observational study to evaluate AB behaviours using a novel bruxism screener (BruxScreen) questionnaire part and Ecological Momentary Assessment (EMA; BruxApp©) on AB behaviours, and to investigate AB's prevalence among masticatory muscle myalgia patients and non-patients	Altogether, 115 participants (masticatory myalgia patients referred to a specialist clinic (n= 67) and non-patients (n= 46)) filled in a bruxism screener questionnaire to report bruxism behaviours and jaw symptoms. A selection of both groups did a week-long EMA (patients n= 12, non-patients n= 11) to report AB behaviours. The	According to BruxScreen, bruxism behaviours and jaw symptoms were more frequent in patients than in non-patients ($p< .001$). Based on EMA, 14.6% of the behaviour in patients was tooth clenching; for non-patients, this was 0.5% ($p< .000$). Relaxed muscles were reported by patients and non-patients at 20.6% and 56.4%,	Awake bruxism behaviours are significantly more frequent in masticatory muscle myalgia patients than non-patients and associate with frequent bruxismrelated symptoms. Self-reported teeth clenching seems to be the most significant sign of AB behaviour.

		chi-squared test was used to determine group differences in categorical variables. A logistic regression model was fitted to study the probability of AB.	respectively (p< .021). Logistic regression, adjusted by age and sex, revealed that patients reported AB 5 times more often than non-patients (OR 4.8, 95% CI 2.1–11.2).	
O'Connor-Reina et al. 2020 **[25]**	A 3-month randomized controlled clinical trial to assess the clinical use of a new mobile health (mHealth) app that uses a smartphone to teach patients with severe obstructive sleep apnea–hypopnea syndrome (OSAHS) to perform oropharyngeal exercises.	Forty patients with severe OSAHS (apnea–hypoxia index [AHI]>30) were enrolled prospectively and randomized into an intervention group that used the app for 90 sessions or a control group. Anthropometric measures, Epworth Sleepiness Scale (0-24), Pittsburgh Sleep Quality Index (0-21), Iowa Oral Performance Instrument (IOPI) scores, and oxygen desaturation index were measured before and after the intervention.	After the intervention, 28 patients remained. No significant changes were observed in the control group; however, the intervention group showed significant improvements in most metrics. AHI decreased by 53.4% from 44.7 (range 33.8-55.6) to 20.88 (14.02-27.7) events/hour (*P*<.001). The oxygen desaturation index decreased by 46.5% from 36.31 (27.19-43.43) to 19.4 (12.9-25.98) events/hour (*P*=.003). The IOPI maximum tongue score increased from 39.83 (35.32-45.2) to 59.06 (54.74-64.00) kPa (*P*<.001), and the IOPI maximum lip score increased from	Orofacial exercises performed using an mHealth app reduced OSAHS severity and symptoms and represent a promising treatment for OSAHS.

			27.89 (24.16-32.47) to 44.11 (39.5-48.8) kPa (*P*<.001). The AHI correlated significantly with IOPI tongue and lip improvements (Pearson correlation coefficient −0.56 and −0.46, respectively; both *P*<.001). The Epworth Sleepiness Scale score decreased from 10.33 (8.71-12.24) to 5.37 (3.45-7.28) in the app group (*P*<.001), but the Pittsburgh Sleep Quality Index did not change significantly.	
Osiewicz et al. 2019 [**26**]	A 1-week observational study to describe the process of translating the smartphone application BruxApp into Polish within the context of an ongoing multicenter project on awake bruxism (AB) epidemiology.	An ongoing cooperation involving 11 universities is based on the adoption of the smartphone-based EMA protocol to collect real time report of AB behaviors in the natural environment. The English version of BruxApp is adopted as a template for the multi-language translation, according to a step-by-step procedure led	: There are two software versions available, viz., BruxApp and BruxApp Research. For both versions, back translation from Polish to English was performed to verify the accuracy of the translation procedure. The validity of the translation has been confirmed by the perfect agreement between the original	As far as clinical studies are concerned, the described strategy to record data can be very useful—patients can acknowledge their habits, monitor changes over time, and implement remedial measures. In the field of research, BruxApp makes it possible to collect and store a huge amount of data about the

		by mother-tongue experts in the field. A dedicated web platform for translation (viz., POEditor) is used. The process of translation into Polish is here described as an example.	and back-translated English versions, and the Polish version of BruxApp can thus be introduced in the clinical and research setting to get deeper into the study of AB epidemiology in Poland	epidemiology of different forms of awake bruxism, both at the individual level and at the population level.
Scheerman et al. 2020 **[27]**	A 12-week randomized controlled clinical trial to evaluate the effectiveness of the WhiteTeeth mobile app, a theory-based mobile health (mHealth) program for promoting oral hygiene in adolescent orthodontic patients.	In this parallel randomized controlled trial, the data of 132 adolescents were collected during three orthodontic check-ups: at baseline (T0), at 6-week follow-up (T1) and at 12-week follow-up (T2). The intervention group was given access to the WhiteTeeth app in addition to usual care (n = 67). The control group received usual care only (n = 65). The oral hygiene outcomes were the presence and the amount of dental plaque (Al-Anezi and Harradine plaque index), and the total number of sites with gingival bleeding	At 6-week follow-up, the intervention led to a significant decrease in gingival bleeding (B = −3.74; 95% CI −6.84 to −0.65) and an increase in the use of fluoride mouth rinse (B = 1.93; 95% CI 0.36 to 3.50). At 12-week follow-up, dental plaque accumulation (B = −11.32; 95% CI −20.57 to −2.07) and the number of sites covered with plaque (B = −6.77; 95% CI −11.67 to −1.87) had been reduced significantly more in the intervention group than in the control group.	The results show that adolescents with fixed orthodontic appliances can be helped to improve their oral hygiene when usual care is combined with a mobile app that provides oral health education and automatic coaching.

		(Bleeding on Marginal Probing Index). Oral health behaviour and its psychosocial factors were measured through a digital questionnaire. We performed linear mixed-model analyses to determine the intervention effects.		
Shirmohammadi et al. 2022 **[28]**	A 3-month randomized controlled clinical trial to assess the effectiveness of a smartphone application promoting preschooler's oral health and to compare it with of common oral health education delivered in paediatric dental settings.	This controlled clinical trial was performed on preschooler–mother dyads referring to the clinic of Tehran School of Dentistry in 2019–2020. Initially, the dyads were randomly partitioned to application intervention or common training groups. The mothers answered an interviewer-administered questionnaire on paediatric dentistry knowledge, attitude and practice regarding children's oral health; modified plaque index (m-PI) and modified gingival index (m-GI) of children were measured.	Among the participants 51 dyad attended baseline and follow-up assessments. The preschoolers mean age was 4.6 ± 1.2 years and 54.4% were girls. Both trainings improved mothers' knowledge and practice regarding children's oral health and reduced children's m-PI and m-GI ($p < 0.050$). The 3-month follow-up revealed a better m-GI in application intervention group ($p < 0.001$)	Considering the greater improvement of paediatric gingival status in the application intervention group, it appears that smartphone applications may provide a promising tool for more prolonged impacts in children oral health care.

		Subsequently, the smartphone application was installed for application intervention group and an educational pamphlet and verbal explanations were given to common training group. In 1-month and 3-month follow-ups, the questionnaires and clinical measurement were re-done. A generalized estimating equation (GEE) was used to investigate the effect of training methods.		
Stanisic et al. 2023 [29]	A 2-week observational study to To translate the application into Swedish, adapt it to Swedish culture and conduct a study to evaluate the usability of the application for studies on family history and associated risk factors.	Translation and cultural adaption of the Swedish version of the application (BruxApp) was carried out in a four-step sequential process. Ten young adults (22–30 years) were recruited together with ten parents (42–67 years) and reported their AB with the application over two seven-day periods. Pain, stress and parafunctional	The back translation check showed minimal discrepancies between the translation and the English version. Participants did not report any problems with the application. Response rates for both groups were 65%. A difference in frequency of AB was shown between young adults and parents (22.0% vs.	The use of application strategies enables data collection on AB which can be used in both clinical and research settings. The results suggest that the Swedish version is ready for implementation and for studies on the relationships between AB, family history and psychosocial factors.

		behaviours were assessed by questionnaires	12.5%, $p < .001$). A positive moderate correlation was found between AB and stress ($r = 0.54$, $p = .017$).	
Zani et al. 2019 [**30**]	A 2-week observational study to discuss the general principles of EMA and EMI (Ecological Momentary Intervention) and comment on a preliminary dataset gathered with a smartphone application in a population of Italian young adults.	A dedicated smartphone application has been used (BruxApp®) on a sample of 30 University students (mean age 24 ± 3.5 years) to record real time report on five specific oral conditions (relaxed jaw muscles, tooth contact, teeth clenching, teeth grinding, mandible bracing) that are related with the spectrum of AB activities. Data were recorded over a 7-day period for two times, with a 1-month interval between the two observation periods. The purpose of collecting data over a second week, 1-month later, was to monitor AB behaviors over time, and test for potential	Over the first 7 days (T1), the average frequency of relaxed jaw muscles reports at the population level was 62%. Teeth contact (20%) and mandible bracing (14%) were the most frequent AB behaviors. No significant gender differences were detected. One month later, during the second week of data collection (T2), the frequency of the conditions was as follows: relaxed jaw muscles 74%, teeth contact 11% and mandible bracing 13%	These data recorded do not allow any generalization due the unrepresentativeness of the study population. On the other hand, they can be used as templates for future comparisons to get deeper into the study of natural fluctuations of AB behaviors as well as into the potential biofeedback effect of an ecological momentary assessment/intervention. It is important to recognize that the use of smartphone technology may help to set range of values for AB frequency in otherwise healthy individuals, in order to stand as comparisons for selected populations with risk or associated factors.

		“EMI” effects.		
Zaror et al. 2019 **[31]**	An observational study to evaluate the validity and usability of a mobile phone-based application for community-based surveillance of traumatic dental injuries.	A mobile phone-based application, Dental Trauma Tracker (DTT), was developed. This system involves a mobile application for general users to report TDIs and a Web application for researchers to generate epidemiological data. The DTT evaluation used mixed methods and was conducted in three phases: (a) validation of a trauma identification system using preselected TDI images; (b) design evaluation by experts; and (c) usability evaluation measured by the reporting of three fictitious TDI cases and using the System Usability Scale (SUS).	In the first phase, 182 participants participated. Most images showed over 95% accuracy, indicating that they adequately represented the type of dentoalveolar trauma being evaluated (κ = 0.75). The design evaluation identified nine usability problems-four of them with a "High priority" to be fixed, four with "Low priority," and one "No fix necessary." A total of 29 volunteers participated in the usability evaluation. The mean time for users to complete all of the reports was 7.8 ± 3.0 minutes. Mean SUS score was 67.4 ± 21.9 (Range: 0-100; worst to best). The global agreement between cases registered with the gold standard was	This preliminary evaluation confirmed the App's usability, using a sample of potential users, as well as reporting on the results of an expert panel review of the DTT. These are the minimum requirements necessary before further expansion and widespread implementation occurs to confirm these results.

			also "Substantial" (κ = 0.71).	
Zolfaghari et al. 2021 **[32]**	A 1-month randomized controlled clinical trial to assess gamified smart phone mobile health application for oral health promotion in early childhood.	In this pretest–posttest controlled clinical trial, a simple app and a gamified version of it were designed to enhance the oral health knowledge and practice of mothers. The app contains information about early childhood caries, health diet, sugars, baby-oral hygiene, fluoride effect, fluoride toothpaste, tooth-brushing training video and regular dental visits. The opinion of experts and 3 mothers were obtained and both apps were revised accordingly. The intervention was implemented on mothers of preschoolers referring to the specialty dental clinic of Tehran School of Dentistry in 2019. The mothers were randomly allocated to the simple app or	Totally, 58 mother and child pairs entered the study; 40% of children were boys. The mean age of children was 4.7 ± 1.2 years. The mean knowledge score of mothers in the pretest was 10.5 and 11.3 in simple app and gamified app group, respectively, which changed to 13.1 and 14.3, respectively in the posttest. The mean practice score of mothers was 4.4 and 4.8 in simple app and gamified app groups, respectively in the pretest, which changed to 8.5 and 8, respectively in the posttest. The mean dental plaque index of children in the pretest was 0.8 and 1 in simple app and gamified app groups, respectively, which changed to 0.5	After 1 month, both apps effectively improved the oral-health knowledge and practice of mothers while oral hygiene as a result of plaque control was superior in children of mothers using the gamified app.

			gamified app group. Before the intervention, all mothers filled out a questionnaire regarding oral health knowledge and practice, and their demographics were collected. The plaque index (PI) of children was also measured. The mothers filled out the same questionnaire 1 month after the intervention, and the PI of children was measured again. Paired *t* test and linear regression model were used for statistical analysis of the data.	and 0.5, respectively in the posttest. Children had better Plaque control in gamified app group ($P < 0.05$).	

Abbreviations: KS: Knowledge Score. OHI-S/GBI: Oral Indexes. OG: Oral Orientation. VG: Video Orientation. m-PI: modified Plaque Index. m-GI: modified Gingival Index. GEE: Generalized Estimating Equation. PI: Plaque Index. OSAHS: Severe Apnea–Hypopnea Sleep Obstructive Syndrome. AHI: Apnea-Hypopnea Index. IOPI: Iowa Oral Performance Instrument. OHEMA: Oral Health Educations using a Mobile App. SWALQoL: Swallowing related Quality of Life. CICP: Community-based Integrated Care Project. GI: Gingival Index. QHI: Quigley Hein Index. OHP: Oral Health Professional. MARS-F: French Mobile App Rating Scale. WADA: WeChat Applet for dental anxiety. SUS: System Usability Scale. DTT: Dental Trauma Tracker. TDI: Traumatic Dental Injuries. BEWE: Basic Erosive Wear Examination Index). EMA: Ecological Momentary Assessment. AB: Awake Bruxism. OHRQoL: Oral Health-Related Quality of Life. OBC: Oral Behaviors Checklist. OR: Odds Ratio. CI: Confidence Interval. EMI: Ecological Momentary Intervention.

Table S6. NHLBI Quality Assessment of Controlled Intervention Studies.

NHLBI Quality Assessment of Controlled Intervention Studies																
**First Author et al., Year**	**Q1**	**Q2**	**Q3**	**Q4**	**Q5**	**Q6**	**Q7**	**Q8**	**Q9**	**Q10**	**Q11**	**Q12**	**Q13**	**Q14**	**Total Score**	**Quality Rating**
Alkadhi et al., 2017 **[12]**	Y	Y	Y	N	Y	N	Y	Y	Y	N	Y	Y	N	Y	10/14	71%
Alkilzy et al., 2019 **[13]**	Y	N	N	N	Y	Y	Y	Y	Y	N	Y	Y	N	Y	9/14	64%
Desai et al., 2021 **[17]**	Y	Y	Y	Y	Y	Y	Y	Y	Y	N	Y	N	N	Y	11/14	79%
Kay et al., 2019 **[20]**	Y	Y	Y	N	N	N	Y	Y	Y	N	Y	N	N	Y	8/14	57%
Ki et al., 2021 **[21]**	Y	N	N	N	N	Y	Y	Y	Y	N	Y	N	N	Y	7/14	50%
Li et al. 2016 **[22]**	Y	Y	Y	N	N	NR	Y	Y	Y	N	Y	Y	Y	Y	10/14	71%
Marchetti et al., 2018 **[23]**	Y	Y	Y	Y	Y	Y	Y	Y	Y	N	Y	Y	N	Y	12/14	86%
O'Connor-Reina et al., 2020 **[25]**	Y	N	Y	Y	Y	Y	N	N	N	N	Y	Y	N	Y	8/14	57%
Scheerman et al., 2020 **[27]**	Y	Y	Y	N	Y	Y	Y	Y	Y	N	Y	N	Y	Y	11/14	79%
Shirmohammadi et al., 2022 **[28]**	Y	Y	Y	N	Y	N	N	Y	Y	N	Y	Y	N	Y	9/14	64%
Zolfaghari et al., 2021 **[32]**	Y	Y	Y	Y	Y	N	Y	Y	Y	N	Y	Y	N	Y	11/14	79%

Q1: Was the study described as randomized, a randomized trial, a randomized clinical trial, or an RCT?, Q2: Was the method of randomization adequate (i.e., use of randomly generated assignment)?, Q3: Was the treatment allocation concealed (so that assignments could not be predicted)?, Q4: Were study participants and providers blinded to treatment group assignment?, Q5: Were the people assessing the outcomes blinded to the participants' group assignments?, Q6: Were the groups similar at baseline on important characteristics that could affect outcomes (e.g., demographics, risk factors, co-morbid conditions)?, Q7: Was the overall drop-out rate from the study at endpoint 20% or lower of the number allocated to treatment?, Q8: Was the differential drop-out rate (between treatment groups) at endpoint 15 percentage points or lower?, Q9: Was there high adherence to the intervention protocols for each treatment group?, Q10: Were other interventions avoided or similar in the groups (e.g., similar background treatments)?, Q11: Were outcomes assessed using valid and reliable measures, implemented consistently across all study participants?, Q12: Did the authors report that the sample size was sufficiently large to be able to detect

a difference in the main outcome between groups with at least 80% power?, Q13: Were outcomes reported or subgroups analyzed prespecified (i.e., identified before analyses were conducted)?, Q14: Were all randomized participants analyzed in the group to which they were originally assigned, i.e., did they use an intention-to-treat analysis?; Total Score: Number of yes; CD: cannot be determined; NA: not applicable; NR: not reported; N: no; Y: yes. Quality Rating: Poor <50%, Fair 50–75%, Good ≥75%.

Table S7. NHLBI Quality Assessment for Cross-Sectional Studies.

NHLBI Quality Assessment of Cross-Sectional Studies																
**First Author et al., Year**	**Q1**	**Q2**	**Q3**	**Q4**	**Q5**	**Q6**	**Q7**	**Q8**	**Q9**	**Q10**	**Q11**	**Q12**	**Q13**	**Q14**	**Total Score**	**Quality Rating**
Câmara-Souza et al. 2020 [**15**]	Y	Y	NR	Y	N	N	Y	Y	Y	Y	Y	N	NR	Y	9/14	64%
Kanoute et al., 2022 [**19**]	Y	Y	NR	N	Y	N	N	Y	Y	N	Y	N	NR	Y	7/14	50%

Q1: Was the research question or objective in this paper clearly stated?, Q2: Was the study population clearly specified and defined?, Q3: Was the participation rate of eligible persons at least 50%?, Q4: Were all the subjects selected or recruited from the same or similar populations (including the same time period)? Were inclusion and exclusion criteria for being in the study prespecified and applied uniformly to all participants?, Q5: Was a sample size justification, power description, or variance and effect estimates provided?, Q6: For the analyses in this paper, were the exposure(s) of interest measured prior to the outcome(s) being measured?, Q7: Was the timeframe sufficient so that one could reasonably expect to see an association between exposure and outcome if it existed?, Q8: For exposures that can vary in amount or level, did the study examine different levels of the exposure as related to the outcome (e.g., categories of exposure, or exposure measured as continuous variable)?, Q9: Were the exposure measures (independent variables) clearly defined, valid, reliable, and implemented consistently across all study participants?, Q10: Was the exposure(s) assessed more than once over time?, Q11: Were the outcome measures (dependent variables) clearly defined, valid, reliable, and implemented consistently across all study participants?, Q12: Were the outcome assessors blinded to the exposure status of participants?, Q13: Was loss to follow-up after baseline 20% or less?, Q14: Were key potential confounding variables measured and adjusted statistically for their impact on the relationship between exposure(s) and outcome(s)?; Total Score: Number of yes; CD: cannot be determined; NA: not applicable; NR: not reported; N: no; Y: yes. Quality Rating: Poor <50%, Fair 50–75%, Good ≥75%.

Table S8. NHLBI Quality Assessment Tool for before–after (pre–post) studies with no control group.

NHLBI Quality Assessment Tool for Before-After (Pre-Post) Studies With No Control Group														
**First Author et al., Year**	**Q1**	**Q2**	**Q3**	**Q4**	**Q5**	**Q6**	**Q7**	**Q8**	**Q9**	**Q10**	**Q11**	**Q12**	**Total Score**	**Quality Rating**
Huang et al., 2022 **[18]**	Y	Y	Y	Y	NR	Y	N	N	Y	N	N	Y	7/12	58%

Q1: Was the study question or objective clearly stated?, Q2: Were eligibility/selection criteria for the study population prespecified and clearly described?, Q3: Were the participants in the study representative of those who would be eligible for the test/service/intervention in the general or clinical population of interest?, Q4: Were all eligible participants that met the prespecified entry criteria enrolled?, Q5: Was the sample size sufficiently large to provide confidence in the findings?, Q6: Was the test/service/intervention clearly described and delivered consistently across the study population?, Q7: Were the outcome measures prespecified, clearly defined, valid, reliable, and assessed consistently across all study participants?, Q8: Were the people assessing the outcomes blinded to the participants' exposures/interventions?, Q9: Was the loss to follow-up after baseline 20% or less? Were those lost to follow-up accounted for in the analysis?, Q10: Did the statistical methods examine changes in outcome measures from before to after the intervention? Were statistical tests done that provided p values for the pre-to-post changes?, Q11: Were outcome measures of interest taken multiple times before the intervention and multiple times after the intervention (i.e., did they use an interrupted time-series design)?, Q12: If the intervention was conducted at a group level (e.g., a whole hospital, a community, etc.) did the statistical analysis take into account the use of individual-level data to determine effects at the group level?; Total Score: Number of yes; CD: cannot be determined; NA: not applicable; NR: not reported; N: no; Y: yes. Quality Rating: Poor <50%, Fair 50–75%, Good ≥75%.

Table S9. NHLBI Quality Assessment Tool for Observational Cohort Studies.

NHLBI Quality Assessment of Observational Cohort Studies															
**First Author et al., Year**	**Q1**	**Q2**	**Q3**	**Q4**	**Q5**	**Q6**	**Q7**	**Q8**	**Q9**	**Q10**	**Q11**	**Q12**	**Q13**	**Q14**	**Total Score**	**Quality Rating**
Butera et al., 2022 **[14]**	Y	Y	NR	Y	N	N	Y	Y	Y	N	Y	NR	NR	Y	8/14	57%
Colonna et al., 2019 **[16]**	Y	Y	NR	Y	N	N	Y	Y	Y	Y	Y	N	NR	Y	9/14	64%
Nykänen et al. 2023 [**24**]	Y	Y	NR	Y	N	N	Y	Y	Y	Y	Y	N	NR	Y	9/14	64%
Osiewicz et al. 2019 [**26**]	Y	Y	NR	Y	N	N	Y	Y	Y	Y	Y	N	NR	Y	9/14	64%
Stanisic et al. 2023 [**29**]	Y	Y	NR	Y	N	N	Y	Y	Y	Y	Y	N	NR	Y	9/14	64%
Zani et al. 2019 [**30**]	Y	Y	NR	Y	N	N	Y	Y	Y	Y	Y	N	NR	Y	9/14	64%
Zaror et al., 2019 **[31]**	Y	Y	NR	NR	Y	Y	Y	Y	Y	N	Y	N	NR	Y	9/14	64%

Q1: Was the research question or objective in this paper clearly stated?, Q2: Was the study population clearly specified and defined?, Q3: Was the participation rate of eligible persons at least 50%?, Q4: Were all the subjects selected or recruited from the same or similar populations (including the same time period)? Were inclusion and exclusion criteria for being in the study prespecified and applied uniformly to all participants?, Q5: Was a sample size justification, power description, or variance and effect estimates provided?, Q6: For the analyses in this paper, were the exposure(s) of interest measured prior to the outcome(s) being measured?, Q7: Was the timeframe sufficient so that one could reasonably expect to see an association between exposure and outcome if it existed?, Q8: For exposures that can vary in amount or level, did the study examine different levels of the exposure as related to the outcome (e.g., categories of exposure, or exposure measured as continuous variable)?, Q9: Were the exposure measures (independent variables) clearly defined, valid, reliable, and implemented consistently across all study participants?, Q10: Was the exposure(s) assessed more than once over time?, Q11: Were the outcome measures (dependent variables) clearly defined, valid, reliable, and implemented consistently across all study participants?, Q12: Were the outcome assessors blinded to the exposure status of participants?, Q13: Was loss to follow-up after baseline 20% or less?, Q14: Were key potential confounding variables measured and adjusted statistically for their impact on the relationship between exposure(s) and outcome(s)?; Total Score: Number of yes; CD: cannot be determined; NA: not applicable; NR: not reported; N: no; Y: yes. Quality Rating: Poor <50%, Fair 50–75%, Good ≥75%.

References

1. Choi EM, Mun SJ, Han SY, Kang JY, Choi JH, Noh HJ. Learning outcomes of a mobile application for dental infection control education. *J Dent Educ*. **2022** Dec;86(12):1678-1684.
2. Golshah A, Dehdar F, Imani MM, Nikkerdar N. Efficacy of smartphone-based Mobile learning versus lecture-based learning for instruction of Cephalometric landmark identification. *BMC Med Educ*. **2020** Aug 31;20(1):287.
3. Humm V, Wiedemeier D, Attin T, Schmidlin P, Gartenmann S. Treatment Success and User-Friendliness of An Electric Toothbrush App: A Pilot Study. *Dent J (Basel)*. **2020** Sep 1;8(3):97.
4. Khatoon B, Hill KB, Walmsley AD. Instant Messaging in Dental Education. *J Dent Educ*. **2015** Dec;79(12):1471-8.
5. Matos Lamenha-Lins R, Maria de Carvelho Pugliesi D, José Camello de Lima F, Regina Oliveira Moreira A, Gonçalves Correia de Leite de Marcelos P, Dos Santos VE Jr. Mobile application as a learning tool for improving dental students' knowledge regarding dental trauma. *Eur J Dent Educ*. **2022** Nov;26(4):700-706.
6. Mergany NN, Dafalla AE, Awooda E. Effect of mobile learning on academic achievement and attitude of Sudanese dental students: a preliminary study. *BMC Med Educ*. **2021** Feb 22;21(1):121.
7. Moylan HB, Carrico CK, Lindauer SJ, Tüfekçi E. Accuracy of a smartphone-based orthodontic treatment-monitoring application: *A pilot study*. *Angle Orthod*. **2019** Sep;89(5):727-733. doi: 10.2319/100218-710.1.
8. Panchal V, Gurunathan D, Shanmugaavel AK. Smartphone application as an aid in determination of caries risk and prevention: A pilot study. *Eur J Dent*. **2017** Oct-Dec;11(4):469-474.
9. Singh RP, Gopalakrishnapillai AC, Bhat N, Pawar A. Perception of Dental and Medical Teaching Faculty Regarding Mobile Dental Application. *J Pharm Bioallied Sci*. **2019** Nov;11(Suppl 3):S530-S539.
10. Stanisic N, Do CT, Skarping S, Chrcanovic B, Bracci A, Manfredini D, Häggman-Henrikson B. Smartphone application to report awake bruxism: Development and testing of the Swedish version and a pilot study to evaluate family history in young adults and their parents: Development and testing of the Swedish version and a pilot study to evaluate family history in young adults and their parents. *J Oral Rehabil*. **2023** May 21.
11. Suner A, Yilmaz Y, Pişkin B. Mobile learning in dentistry: usage habits, attitudes and perceptions of undergraduate students. *PeerJ*. **2019** Jul 29;7:e7391.
12. Alkadhi OH, Zahid MN, Almanea RS, Althaqeb HK, Alharbi TH, Ajwa NM. The effect of using mobile applications for improving oral hygiene in patients with orthodontic fixed appliances: a randomised controlled trial. *J Orthod*. **2017** Sep;44(3):157-163.
13. Alkilzy M, Midani R, Höfer M, Splieth C. Improving Toothbrushing with a Smartphone App: Results of a Randomized Controlled Trial. *Caries Res*. **2019**;53(6):628-635.
14. Butera A, Maiorani C, Gallo S, Pascadopoli M, Buono S, Scribante A. Dental Erosion Evaluation with Intact-Tooth Smartphone Application: Preliminary Clinical Results from September 2019 to March 2022. *Sensors (Basel)*. **2022** Jul 8;22(14):5133.
15. Câmara-Souza MB, Carvalho AG, Figueredo OMC, Bracci A, Manfredini D, Rodrigues Garcia RCM. Awake bruxism frequency and psychosocial factors in college preparatory students. *Cranio*. **2023** Mar;41(2):178-184.
16. Colonna A, Lombardo L, Siciliani G, Bracci A, Guarda-Nardini L, Djukic G, Manfredini D. Smartphone-based application for EMA assessment of awake bruxism: compliance evaluation in a sample of healthy young adults. *Clin Oral Investig*. **2020** Apr;24(4):1395-1400.

17. Desai RV, Badrapur NC, Mittapalli H, Srivastava BK, Eshwar S, Jain V. "BRUSH UP": AN INNOVATIVE TECHNOLOGICAL AID FOR PARENTS TO KEEP A CHECK OF THEIR CHILDREN'S ORAL HYGIENE BEHAVIOUR. *Rev Paul Pediatr.* **2021** Apr 2;39:e2020085.
18. Huang X, Zeng J, Zhao N, Fan L, Ruan D, Wang J, Hong X, Yu C. Experience of using a smartphone WeChat applet for dental anxiety assessment and preoperative evaluation: A nationwide multicenter study. *Front Public Health.* **2022** Jul 18;10:900899.
19. Kanoute A, Carrouel F, Gare J, Dieng SN, Dieng A, Diop M, Faye D, Fraticelli L, Bourgeois D. Evaluation of Oral Hygiene-Related Mobile Apps for Children in Sub-Saharan Africa. *Int J Environ Res Public Health.* **2022** Oct 1;19(19):12565.
20. Kay E, Shou L. A randomised controlled trial of a smartphone application for improving oral hygiene. *Br Dent J.* **2019** Apr;226(7):508-511.
21. Ki JY, Jo SR, Cho KS, Park JE, Cho JW, Jang JH. Effect of Oral Health Education Using a Mobile App (OHEMA) on the Oral Health and Swallowing-Related Quality of Life in Community-Based Integrated Care of the Elderly: A Randomized Clinical Trial. *Int J Environ Res Public Health.* **2021** Nov 7;18(21):11679.
22. Li X, Xu ZR, Tang N, Ye C, Zhu XL, Zhou T, Zhao ZH. Effect of intervention using a messaging app on compliance and duration of treatment in orthodontic patients. *Clin Oral Investig.* **2016** Nov;20(8):1849-1859.
23. Marchetti G, Fraiz FC, Nascimento WMD, Soares GMS, Assunção LRDS. Improving adolescents' periodontal health: evaluation of a mobile oral health App associated with conventional educational methods: a cluster randomized trial. *Int J Paediatr Dent.* **2018** Jul;28(4):410-419.
24. Nykänen L, Manfredini D, Lobbezoo F, Kämppi A, Bracci A, Ahlberg J. Assessment of awake bruxism by a novel bruxism screener and ecological momentary assessment among patients with masticatory muscle myalgia and healthy controls. *J Oral Rehabil.* **2023** Apr 10.
25. O'Connor-Reina C, Ignacio Garcia JM, Rodriguez Ruiz E, Morillo Dominguez MDC, Ignacio Barrios V, Baptista Jardin P, Casado Morente JC, Garcia Iriarte MT, Plaza G. Myofunctional Therapy App for Severe Apnea-Hypopnea Sleep Obstructive Syndrome: Pilot Randomized Controlled Trial. *JMIR Mhealth Uhealth.* **2020** Nov 9;8(11):e23123.
26. Osiewicz MA, Lobbezoo F, Bracci A, Ahlberg J, Pytko-Polończyk J, Manfredini D. Ecological Momentary Assessment and Intervention Principles for the Study of Awake Bruxism Behaviors, Part 2: Development of a Smartphone Application for a Multicenter Investigation and Chronological Translation for the Polish Version. *Front Neurol.* **2019** Mar 5;10:170.
27. Scheerman JFM, van Meijel B, van Empelen P, Verrips GHW, van Loveren C, Twisk JWR, Pakpour AH, van den Braak MCT, Kramer GJC. The effect of using a mobile application ("WhiteTeeth") on improving oral hygiene: A randomized controlled trial. *Int J Dent Hyg.* **2020** Feb;18(1):73-83.
28. Shirmohammadi M, Razeghi S, Shamshiri AR, Mohebbi SZ. Impact of smartphone application usage by mothers in improving oral health and its determinants in early childhood: a randomised controlled trial in a paediatric dental setting. *Eur Arch Paediatr Dent.* **2022** Aug;23(4):629-639.
29. Stanisic N, Do CT, Skarping S, Chrcanovic B, Bracci A, Manfredini D, Häggman-Henrikson B. Smartphone application to report awake bruxism: Development and testing of the Swedish version and a pilot study to evaluate family history in young adults and their parents: Development and testing of the Swedish version and a pilot study to evaluate family history in young adults and their parents. *J Oral Rehabil.* **2023** May 21.
30. Zani A, Lobbezoo F, Bracci A, Ahlberg J, Manfredini D. Ecological Momentary Assessment and Intervention Principles for the Study of Awake Bruxism Behaviors, Part 1: General Principles and Preliminary Data on Healthy Young Italian Adults. *Front Neurol.* **2019** Mar 1;10:169.
31. Zaror C, Espinoza-Espinoza G, Atala-Acevedo C, Muñoz-Millán P, Li Y, Clarke K, Onetto J, Díaz J, Hallet K, Manton D, Mariño R. Validation and usability of a mobile phone application for epidemiological surveillance of traumatic dental injuries. *Dent Traumatol.* **2019** Feb;35(1):33-40.
32. Zolfaghari M, Shirmohammadi M, Shahhosseini H, Mokhtaran M, Mohebbi SZ. Development and evaluation of a gamified smart phone mobile health application for oral health promotion in early childhood: a randomized controlled trial. *BMC Oral Health.* **2021** Jan 7;21(1):18.
